# MFNet: Meta‐learning based on frequency‐space mix for MRI segmentation in nasopharyngeal carcinoma

**DOI:** 10.1111/jcmm.18355

**Published:** 2024-04-29

**Authors:** Yin Li, Qi Chen, Hao Li, Song Wang, Nutan Chen, Ting Han, Kai Wang, Qingqing Yu, Zhantao Cao, Jun Tang

**Affiliations:** ^1^ Department of Otorhinolaryngology The First People's Hospital of Foshan Foshan China; ^2^ Department of Radiology The Second Affiliated Hospital of Anhui Medical University Hefei China; ^3^ Department of Infectious Diseases, The First People's Hospital of Changde City, Xiangya School of Medicine Central South University Changde China; ^4^ University of Electronic Science and Technology of China Chengdu China; ^5^ Machine Learning Research Lab, Volkswagen Group Munich Germany; ^6^ Department of Radiology The First People's Hospital of Foshan Foshan China; ^7^ Department of Research CETC Cyberspace Security Technology CO., LTD. Chengdu China

**Keywords:** data augmentation, domain generalization, feature mix, image segmentation, nasopharyngeal carcinoma

## Abstract

Deep learning techniques have been applied to medical image segmentation and demonstrated expert‐level performance. Due to the poor generalization abilities of the models in the deployment in different centres, common solutions, such as transfer learning and domain adaptation techniques, have been proposed to mitigate this issue. However, these solutions necessitate retraining the models with target domain data and annotations, which limits their deployment in clinical settings in unseen domains. We evaluated the performance of domain generalization methods on the task of MRI segmentation of nasopharyngeal carcinoma (NPC) by collecting a new dataset of 321 patients with manually annotated MRIs from two hospitals. We transformed the modalities of MRI, including T1WI, T2WI and CE‐T1WI, from the spatial domain to the frequency domain using Fourier transform. To address the bottleneck of domain generalization in MRI segmentation of NPC, we propose a meta‐learning approach based on frequency domain feature mixing. We evaluated the performance of MFNet against existing techniques for generalizing NPC segmentation in terms of Dice and MIoU. Our method evidently outperforms the baseline in handling the generalization of NPC segmentation. The MF‐Net clearly demonstrates its effectiveness for generalizing NPC MRI segmentation to unseen domains (Dice = 67.59%, MIoU = 75.74% T1W1). MFNet enhances the model's generalization capabilities by incorporating mixed‐feature meta‐learning. Our approach offers a novel perspective to tackle the domain generalization problem in the field of medical imaging by effectively exploiting the unique characteristics of medical images.

## 1 INTRODUCTION

Nasopharyngeal carcinoma (NPC) is a highly aggressive malignant tumour that originates in the nasopharynx and pathologically classified into keratinized squamous cell carcinoma and non‐keratinized carcinoma.^1^ It is estimated that approximately 130,000 new cases of NPC occurred worldwide in 2018, with particularly high incidence rates in Southern China, Southeast Asia, and North Africa.^2^ Southern China is known to have one of the highest NPC incidence rates, with up to 50 cases per 100,000 individuals.^3^ Radiotherapy is the primary treatment for NPC. Considering the adjacent critical structures in nasopharynx, accurately defining the radiation field is essential to achieve optimal therapeutic efficacy while minimizing local complications. The success of NPC radiotherapy significantly depends on the accurate characterization of the tumour volume and its precise boundaries, which are used to formulate dosimetric parameters while sparing uninvolved critical structures. Traditionally, manual delineation of the nasopharynx gross tumour volume (GTVnx) using computed tomography (CT) imaging by experienced radiologists has been the standard procedure for NPC treatment planning. However, magnetic resonance imaging (MRI) provides valuable information for identifying the tumour extent, volume, and invasion of adjacent structures. MRI has the advantage of better boundary distinction compared to PET‐CT and CT imaging modalities.^4^

In recent years, deep convolutional neural networks have made significant strides in medical image segmentation, greatly enhancing the accuracy of this technique. Ideally, when training datasets encompass a large number of high‐quality images from various medical centres utilizing different imaging vendors and protocols, highly generalizable models can be achieved in the field of medical imaging. However, NPC presents unique challenges that make it difficult to obtain a large‐scale training dataset from diverse hospitals. These limitations result in small training datasets lacking the necessary diversity, which ultimately hinders the models' ability to perform consistently on data from ‘unseen’ domains. For instance, while a deep learning model might achieve an error rate of 5.5% for retinal image analysis using images from the same vendor as those in the training dataset, this error rate could increase to 46.6% when using images from a different vendor.^5^ Consequently, this lack of generalizability has emerged as a significant obstacle in the practical implementation of deep learning models in clinical practice.^6^

Currently, there are three popular solutions proposed in the industry to address the issue of poor generalization of models in unseen domains. The first solution is transfer learning, where a pre‐trained network is fine‐tuned using a small amount of labelled data from the unseen domain.^7^ While this approach shows promising performance, it requires retraining the new model before deployment, making it impractical for different patient populations (e.g., multiple clinical centres) or unpredictable scenarios (e.g., rural areas). The second solution involves domain adaptation using multiple‐source data, with one of the datasets serving as the unseen domain. By utilizing multiple datasets with different distributions, meta‐learning strategies simulate the training–testing process of domain generalization during model optimization.^8^, ^9^, ^10^ However, in the NPC, collecting multi‐source data is challenging due to the regional discrepancy of the incidence rate. The third solution is data augmentation in a single domain, with various complex techniques being employed to expand the coverage of data distribution. Specifically, additional training data samples are generated in the image domain,^11^ semantic space^12^ or through adversarial learning.^13^ Data augmentation has been proven to be one of the most important regularization techniques related to the generalization performance of deep learning models.^14^ It helps prevent overfitting to the training data and enables better generalization to the test data. To the best of our knowledge, there is currently no comprehensive investigation on single‐domain generalization for NPC MRI images, and no optimization‐related research has been conducted specifically for addressing the domain generalization problem in the context of NPC.

MRI technology performs a *z*‐direction selection after receiving a signal. Then, it utilizes the Fourier transform to convert the phase spectrum and frequency spectrum into a spatial domain. Visual psychophysics research has revealed that the low‐level distribution (i.e., style) and high‐level semantics of an image can be captured through the amplitude spectrum and phase spectrum in the frequency domain.^5^, ^15^ Hong et al.^16^ have demonstrated in their style‐mixing approach that the low‐level distribution (i.e., style) can enhance the generalization capability of neural networks. Inspired by this, in our work, we believe that mixing the low‐level distributions from two input images can facilitate the creation of more valuable samples, thereby ultimately enhancing the generalization ability of the model. Based on the characteristics of MRI, we have designed a continuous frequency space interpolation mix mechanism. Furthermore, we have adopted meta‐learning on the high‐level semantics to train the network. This mechanism helps the network acquire domain invariance from the feature distribution space, thus enhancing the network's generalization in the unseen domain.

We list the contributions of our work as follows:

1. For the purpose of analysing and evaluating the performance of existing single‐domain generalization methods in NPC image segmentation, we collected two new datasets. These datasets include 44 NPC patients obtained at The Second Affiliated Hospital of Anhui Medical University and 277 NPC patients at The First People's Hospital of Foshan, China, along with their respective MRI images, which have been manually annotated by experienced oncologists.
2. For the task of single‐domain segmentation generalization, we employ state‐of‐the‐art domain generalization methods for medical image segmentation of NPC images to identify the lesion areas. We perform a systematic evaluation of these methods on a newly collected dataset, establishing a benchmark. Our research has the potential to benefit other researchers in the same domain.
3. We present a novel MF‐Net model that tackles the formidable task of domain generalization in MRI segmentation for NPC. This meta‐learning approach, based on frequency‐space mix, enables the model to effortlessly segment images in target domains that have never been encountered before, eliminating the need for cost‐consuming retraining.
4. Our method exhibits compelling efficacy, as substantiated by an extensive array of experiments. On the dataset, we have meticulously curated, our approach surpasses the prevailing domain generalization methods, setting a new standard in the field. Furthermore, we conduct a rigorous analysis, meticulously scrutinizing the performance of various methodologies.

## 2 MATERIALS AND METHODS

In clinical practice, MRI scans of patients with NPC typically comprise of T1‐weighted, T2‐weighted and contrast‐enhanced T1‐weighted images. These three modalities in MRI exhibit distinct signal intensities for tissue. Among them, T1‐weighted imaging employs a shortened repetition time (TR) and echo time (TE) to evaluate spin lattice relaxation, thereby providing enhanced visualization of anatomical structures due to its preference for low water content and high fat content signals. In contrast, T2‐weighted imaging employs longer TR and TE to measure spin relaxation, emphasizing high water content signals and minimizing fat content signals, which enables better observation of lesions. By injecting a gadolinium contrast agent, contrast‐enhanced T1‐weighted imaging induces T1 shortening effect, enhancing signal strength and predominantly reflecting the microvessels within the tissue. This technique is often utilized for analysing blood vessels generated by lesions. Consequently, T1‐weighted, T2‐weighted and contrast‐enhanced T1‐weighted imaging contributes to the comprehensive assessment of the anatomical structure, lesions and microvessels in NPC. They mutually complement each other in revealing the primary lesion of NPC, lymph nodes and adjacent tissues. Radiologists typically diagnose NPC and its surrounding tissues by carefully inspecting the three MRI modalities. However, current domain generalization methods for MRI tumour segmentation fail to account for the specific characteristics of medical images. To address domain generalization in NPC MRI tumour segmentation, we have devised a network model named MF‐Net, which effectively assimilates the information from the aforementioned distinct MRI parameters. In the subsequent section, we will comprehensively introduce the MF‐Net approach.

### 2.1 Preliminaries

To address the domain generalization issue in NPC MRI image segmentation, we aim to blend the feature distributions and learn parameters that exhibit superior generalization. For this purpose, we transform the modalities of nasopharyngeal carcinoma patients' MRIs, including T1WI, T2WI and CE‐T1WI, from the spatial domain to the frequency domain using Fourier transform. Fourier transform can effectively separate the frequency components of a signal.^17^

Frequency‐space mixing manipulates data in the frequency domain, prioritizing intrinsic patterns rather than pixel values. This approach is supported by advancements in deep learning, which have shown that neural networks are adept at manipulating and interpreting data in the frequency domain.^18^ The frequency domain consists of the phase spectrum and the magnitude spectrum, where the low‐level distribution of the image can be captured by the magnitude in frequency space. By blending and enhancing the magnitude spectra of different modalities, we aim to improve the model's generalization while preserving the unaltered phase spectra.

We define $D=\left\{D^{S},D^{U}\right\}$ to represent NPC data from two different hospitals. $D^{S}$ represents the visible domain used to train the model $M_{\theta }$, while $D^{U}$ represents the invisible domain consisting of data from other hospitals that were not involved in the training. The differences between these two domains arise from variations in patients, geographical factors, equipment and medical practitioners' imaging techniques.

In the context of this study, $D^{S}={\left\{x_{i}^{k},y_{i}^{k}\right\}}_{i=1}^{N}$ denotes the ensemble of source domain images. Here, k∈K indicates that each patient's images encompass of *K* modalities. $x_{i}^{k}\in R^{W\times H\times C}$ signifies the image representing modality k in the MRIs of patient i, where $y_{i}^{k}$ represents the segmentation label corresponding to $x_{i}^{k}$. *W*, *H* and *C* denote the width, height and number of channels of the image, respectively. *N* corresponds to the total number of available samples.

This investigation aims to train a segmentation model, denoted as $M_{\theta }$, using the source domain $D^{S}$ to attain robust generalization on the unseen domain $D^{U}$.

### 2.2 Feature mix in frequency space

To address the domain generalization issue in NPC MRI image segmentation, we aim to blend the feature distributions and learn parameters that exhibit superior generalization. For this purpose, we transform the modalities of NPC patients' MRIs, including T1WI, T2WI and CE‐T1WI, from the spatial domain to the frequency domain using Fourier transform. The frequency domain consists of the phase spectrum and the magnitude spectrum, where the magnitude in frequency space can capture the low‐level distribution of the image. By blending and enhancing the magnitude spectra of different modalities, we aim to improve the model's generalization while preserving the unaltered phase spectra, which encapsulate the fundamental semantics of the image.

For a sample $x_{i}^{k}$, we employ the Fast Fourier Transform^19^ to convert it into the frequency domain:

$$F\left(x_{i}^{k}\right)=A_{i}^{k}+P_{i}^{k}$$

where $A_{i}^{k}$ corresponds to the amplitude spectrum of sample $x_{i}^{k}$, while $P_{i}^{k}$ represents the phase spectrum. Notably, the amplitude spectrum captures the intricacies of low‐level distributions within an image, whereas the phase spectrum conveys rich and meaningful high‐level semantics.

Thereafter, we will interpolate and mix the amplitude spectrum according to a ratio λ:

$$A_{i}^{m}=\left(1-\text{λ}\right)A_{i}^{k}+\text{λ}A_{i}^{h},$$

where h∈K, k≠h. In our approach, the interpolation rate λ ranges from 0.0 to 1.0. Following the acquisition of $A_{i}^{m}$, we employ an inverse Fourier transform to procure the amalgamated image $x_{i}^{m}$. The inverse Fourier transform formula is outlined as

$$x_{i}^{m}=F^{-1}\left(A_{i}^{m},P_{i}^{k}\right).$$

### 2.3 Meta‐learning based on mixed feature

Our objective is to acquire a profound understanding of the fundamental characteristics inherent in NPC MRI data, ultimately bolstering the model's capacity to generalize in uncharted territories. The meta‐learning algorithm can remarkably adapt and internalize the most optimal learning strategy autonomously. Within our framework, we have original images and mixed images. To harness the full potential of these two image types and elevate the overall generalizability of the nasopharyngeal cancer image segmentation model, we have devised the meta‐learning based on mixed‐feature approach. In this approach, the generalization across domains is accomplished by harnessing data mixtures originating from dissimilar distributions in the frequency domain.

To provide a more concise and elucidating description of our method, we introduce the notion of the original dataset as $D_{\text{init}}^{S}$ and the mixed dataset as $D_{\mathrm{mix}}^{S}$. Moreover, we define a set of tasks as $T=\left\{T_{1},T_{2}\right\}$, where $T_{1}$ corresponds to the original dataset $D_{\text{init}}^{S}$, and $T_{2}$ corresponds to the mixed dataset $D_{\mathrm{mix}}^{S}$. We define the model corresponding to $T_{1}$ as $f_{\theta }$, with the model's parameters described by θ.

Initially, we initialize the parameters θ with random values. Subsequently, we proceed to train the network on the designated training set $D_{\text{train}}^{S}$ for the $T_{1}$ task. Throughout this process, we optimize the network by utilizing the dice loss function:

$$L_{\text{init}}=L\left(f_{\theta }\left(x_{i}^{k}\right)\right)=1-\frac{2\sum_{i=1}^{N}p_{i}g_{i}}{\sum_{i=1}^{N}p_{i}^{2}+\sum_{i=1}^{N}g_{i}^{2}},$$

where N represents the total number of pixels, $p_{i}$ denotes the model's prediction for the i‐th pixel, and $g_{i}$ represents the true label value of the i‐th pixel. It reaches its minimum value of 0 when the predicted and true results are perfectly aligned and its maximum value of 1 when they are entirely discordant. In contrast to the cross‐entropy loss function, the dice loss function addresses class imbalance issues by incorporating the weight of each pixel in its computation rather than relying solely on pixel count as the weight. This renders Dice loss particularly well‐suited for mitigating class imbalance challenges in segmentation tasks.

Subsequently, we employ the gradient descent algorithm to minimize the loss and obtain the parameters $\hat{\theta }$ that yield a relatively optimal solution:

$$\hat{\theta }=\theta -\gamma {▽}_{\theta }L\left(f_{\theta }\left(x_{i}^{k}\right)\right),$$

where $\hat{\theta }$ represents the optimal parameters for the task, γ is a hyperparameter, and L represents the result of gradient computation.

Before proceeding to the next batch of tasks, we implement a meta‐update optimization strategy to train task $T_{2}$. First, we obtain a relatively optimal parameter $\hat{\theta }$ based on the previous step, and in the training of tasks in this batch, we train task $T_{2}$ based on this parameter, which can reduce the number of gradient descent steps. The mixed loss function is defined as:

$$L_{\mathrm{mix}}=L\left(f_{\hat{\theta }}\left(x_{i}^{m}\right)\right),$$

where the parameter $\hat{\theta }$ represents the initialization parameter obtained through task $T_{1}$ and used for task $T_{2}$.

### 2.4 Learning MFNet

The objective function of our model is composed of two components:

$$\min_{\theta ,\hat{\theta }}\;L=\alpha L_{\text{init}}+\beta L_{\mathrm{mix}},$$

where α and β are trade‐off coefficients, modulating the significance of their respective terms in the model. The model is trained by optimizing the objective function L with stochastic gradient descent algorithms.

## 3 RESULTS

**FIGURE 1:**
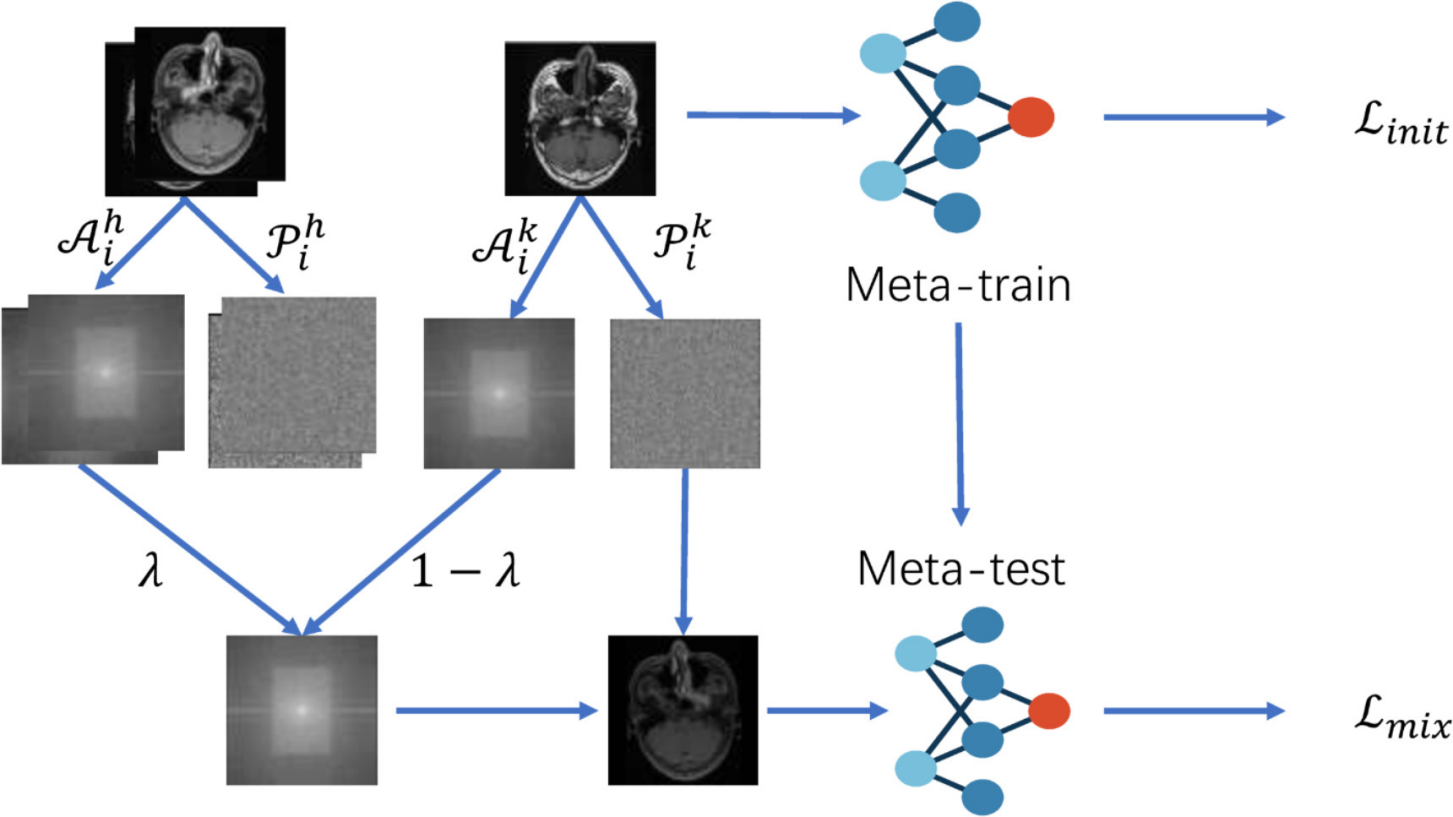
The overall architecture of our proposed MF‐Net. Given an MRI image of nasopharyngeal carcinoma, we employ Fourier transform to shift the MRI's morphology from the spatial domain to the frequency domain. We blend and enhancing the magnitude spectra of different modalities while preserving the phase spectrum, which encapsulates the image's fundamental semantics. We propose a meta‐learning method based on mixed features to acquire a comprehensive understanding of the inherent fundamental features within nasopharyngeal carcinoma MRI data, ultimately enhancing the model's generalization capability in unexplored territories.

We provide a comprehensive review of the experiments and the corresponding results. We show the importance of these experiments in validating the effectiveness and real‐world applicability of the MFNet model (Figure 1). This segment is essential for demonstrating how the model performs under various conditions and its potential impact in the field of MRI segmentation for NPC.

### 3.1 Dataset

Collecting a well‐defined dataset is key to the research on domain generalization of NPC segmentation. To this end, our research endeavours have entailed fruitful collaborations with two esteemed medical institutions, where we meticulously gathered data from patients diagnosed with NPC and who underwent treatment between the esteemed period of July 2013 and January 2022. The first dataset consists of NPC MRIs from 44 patients obtained at The Second Affiliated Hospital of Anhui Medical University. These data were captured using a Siemens MAGNETOM Verio 3T device, with Gadobenate Dimeglumine Injection as the contrast agent. The second dataset consists of NPC MRIs from 277 patients at The First People's Hospital of Foshan, China.^20^ These data were captured using a GE Discovery MR750w 3.0T and Philips Achieva 1.5T devices, with Gadoteric Acid Meglumine Salt Injection as the contrast agent. The number of slices varies from patient to patient, from 15 to 38. It is important to acknowledge that our data collection process rigorously adhered to specific criteria, leading to the exclusion of certain cases that would compromise the integrity of our dataset. Specifically, patients who had undergone prior radiation therapy or chemotherapy, which could potentially distort the internal structure of the tumour and compromise the reliability of lesion boundaries, were purposefully excluded. Similarly, individuals with histories of other malignancies were excluded to ensure the dataset's homogeneity. Finally, images that failed to meet predetermined criteria, such as insufficient coverage of the lesion areas, inadequate image resolution or the presence of artefacts, were diligently filtered out to maintain the dataset's quality and reliability.

**FIGURE 2:**
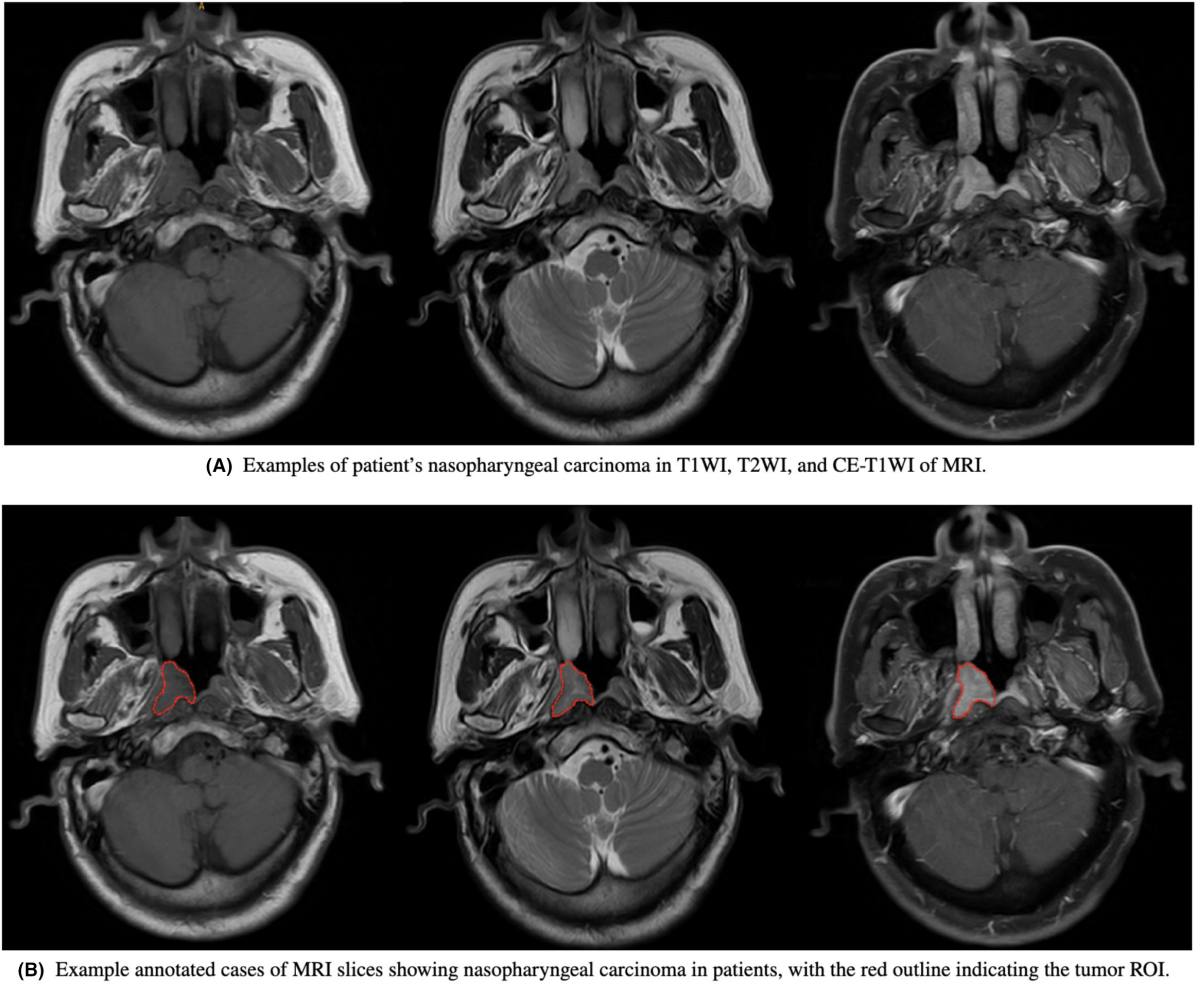
An example of MRI slices from a patient with nasopharyngeal carcinoma, with T1WI, T2WI, and CE‐T1WI images arranged from left to right.

After transferring MRI images into the radiotherapy target volume delineation system MIM Software (Beijing Co., Ltd.), two experienced radiation oncologists with more than 10 years of experience in head and neck cancer GTV in transverse T1WI, T2WI and CE‐T1WI, respectively. When disagreements occurred during the contouring process, a third researcher stepped in to resolve the disagreements by discussions. For better performance and convenience, the format of images was converted from DICOM format into JPEG format, and the contours of lesions were transformed into binary masks and coordinates of bounding boxes. All the above steps were performed in SimpleITK and OpenCV. The annotated data examples are shown in Figure 2.

In our scenario, the data from The First People's Hospital of Foshan, China, are used as the training set, which is randomly split into 80% for training and 20% for validation. Due to the regional characteristics and differences in capturing devices and contrast agents, the data from The Second Affiliated Hospital of Anhui Medical University belong to an unknown domain compared to The First People's Hospital of Foshan, China. In this paper, we define it as the test set. To evaluate fairness, we selected slices from each patient which contained tumour.

### 3.2 Implementation

The experiments described in this paper are all based on the Pytorch deep learning framework. Our code is released at https://github.com/caozhantao/MFNet.git. The GPU used for the experiments is Tesla M40, and the operating system is Ubuntu 22.04.3 LTS. For the sake of fairness, UNet is used as the base network for all comparative experiments. During the experiment, the SGD optimizer is used as the optimizer during the training phase.^21^ To adapt to the input of the UNet network, the image size is resized to 512×512. The experiment utilized a polynomial learning rate scheduler. The momentum value is set to 0.9, the weight decay value is set to 5e−4, and the batch size is set to 4. In the comparative experiments, all experiments are conducted for 30 iterations to obtain convergent results. To ensure the reproducibility of the experiment, we employed five independent random number seeds in our study. We conducted experiments for each seed and ultimately obtained the average of the results from five experiments.

### 3.3 Evaluation metrics

We adopt three popularly used evaluation metrics, dice coefficient (Dice) and mean intersection over union (MIoU), which are defined as:

$$\begin{array}{r} \text{Dice}=\frac{1}{N}\sum_{i=1}^{N}\frac{2\left|X_{i}^{\mathrm{gt}}\cap X_{i}^{\text{pred}}\right|}{\left|X_{i}^{\mathrm{gt}}\right|+\left|X_{i}^{\text{pred}}\right|},\\ \text{MIoU}=\frac{1}{N}\sum_{i=1}^{N}\frac{\left|X_{i}^{\mathrm{gt}}\cap X_{i}^{\text{pred}}\right|}{\left|X_{i}^{\mathrm{gt}}\cup X_{i}^{\text{pred}}\right|}, \end{array}$$

where N is the number of images, $X_{i}^{\mathrm{gt}}$ is the ground truth lesion region, $X_{i}^{\text{pred}}$ is the predicted lesion region, and d is the mean distances between the surface voxel of binary objects in $X_{i}^{\text{pred}}$ and their nearest partner surface voxel of a binary object in $X_{i}^{\mathrm{gt}}$. The higher Dice and MIoU show a higher overlapped rate between the prediction and the true lesion region.

### 3.4 Experimental

To demonstrate the effectiveness of MF‐Net, we conduct extensive experiments over the empirical data that we collect. Our method is compared with the existing state‐of‐the‐art works on handling domain generalization, which are detailed as follows:

1. UNet: The base model of this work.
2. Mixup: Train the neural network to perform linear interpolation between two random training examples and their labels. Mixup encourages the neural network to exhibit simple linear behaviour between training images, ultimately improving its generalization ability.^22^
3. StyleMix: Stylemix^16^ aims to separate the style and content of images and mix them separately, leading to the generation of higher‐quality mixed images. This is achieved by utilizing the AdaIN model, a popular approach for style transfer, to separate the style and content components of the images. The differences between the style and content are then fused independently, resulting in more effective and sophisticated image mixing.
4. MI‐SegNet: Bi et al. designed a segmentation network based on mutual information,^23^ which extracts style (image appearance) and anatomy (shape) features from ultrasound images. The network generates segmentation masks based on anatomical features, effectively excluding domain‐related features. This allows the segmentation network to understand the statistical shape model of the target anatomical structure and generalize it to different unknown scenarios.
5. Dual‐Norm: Zhou et al. proposed a novel approach to address the challenge of domain generalization in unknown target domains.^24^ Firstly, the model applies nonlinear transformations to enhance the similarity and dissimilarity between source and target images. Then, it employs a dual normalization approach. Finally, a style‐based selection scheme is employed to automatically select the suitable pathway during the testing phase.
6. MF‐Net: Meta‐learning based on frequency‐space mix for MRI segmentation in NPC proposed in this work.

Our method utilized UNet as the backbone architecture to ensure fairness and eliminate bias. Among the comparative methods, MI‐SegNet employed its own designed network, while the remaining approaches used UNet as their basic model. This choice of backbone architecture ensured a consistent starting point for all methods, allowing for a fair and objective comparison.

We first evaluated the generalization performance of various existing medical image segmentation methods (including the UNet, BigAug, MI‐SegNet and Dual‐Norm methods) on NPC MRIs through extensive experiments. The evaluation metrics used include Dice and MIoU, with cross‐validation performed for the three signal types: T1WI, T2WI and CE‐T1WI. From the results in Table 1, we can observe that the first row represents our base Net, the second row represents BigAug, which enhances the data in 3D and slightly improves efficiency. The third row represents MI‐SegNet, which performs poorly due to its segmentation mask being generated based on anatomical features, enabling the segmentation network to understand the statistical shape of the target anatomy. However, this design is specific to ultrasound images, and the significant variations in anatomical characteristics across different types of images make it difficult to apply this method to other medical images. The fourth row represents Mixup, a data mixing method that directly combines the data. While it improves generalization performance in natural scene classification scenarios, applying this method in medical imaging increases tumour regions' noise and imprecise segmentation. Dual‐Norm demonstrates relatively good performance by enhancing the images with two sets of augmentations: one set with similar sources and another with dissimilar sources, preserving the style information of both domains. It retains the best normalization path by comparing and selecting the optimal style, thus exhibiting good generalization across different medical images.

**TABLE 1:** Comparisons with the existing state‐of‐the‐art works on domain generalization in terms of Dice and MIoU from our dataset.

Model	T1WI		T2WI		CE‐T1WI	
	Dice (%)	MIoU (%)	Dice (%)	MIoU (%)	Dice (%)	MIoU (%)
UNet^25^	64.96	75.38	47.38	65.74	59.91	72.85
Mixup^22^	63.26	74.28	47.78	66.93	60.36	74.11
StyleMix^16^	64.59	75.02	48.09	65.91	60.69	73.95
MI‐SegNet^23^	35.08	47.88	27.03	45.97	32.02	47.34
Dual‐Norm^24^	65.09	73.80	46.13	64.30	63.49	73.55
MF‐Net	67.59	75.74	52.28	67.66	65.72	74.76

**FIGURE 3:**
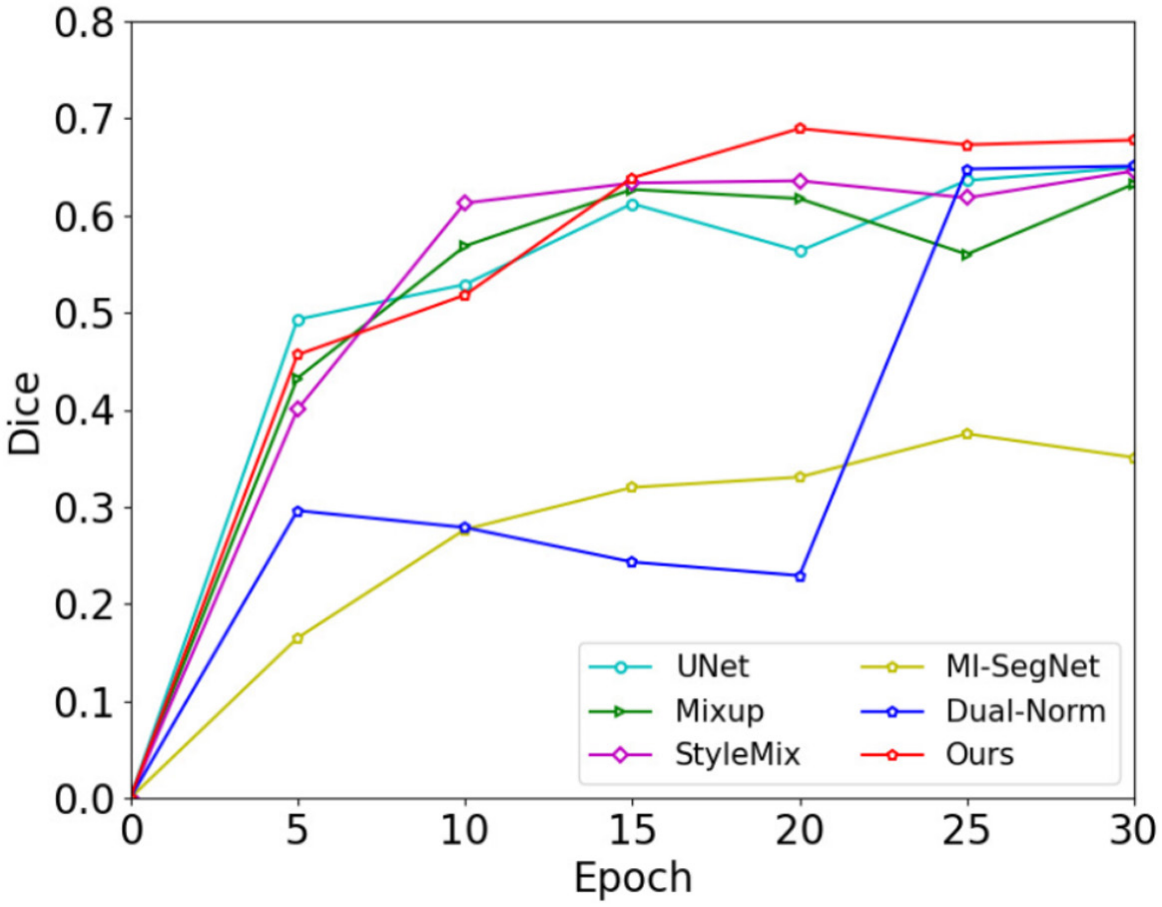
The test dice of T1WI.

**FIGURE 4:**
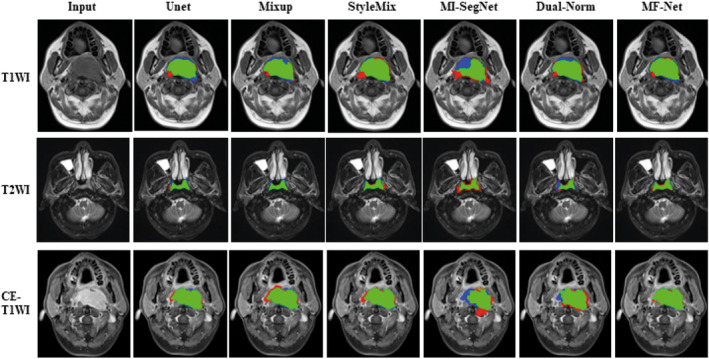
Segmentation using different segmentation methods with multiple medical imaging modalities in various medical cases. The green part represents the correct segmentation area, while the red and blue parts represent the over‐segmentation and under‐segmentation, respectively.

**FIGURE 5:**
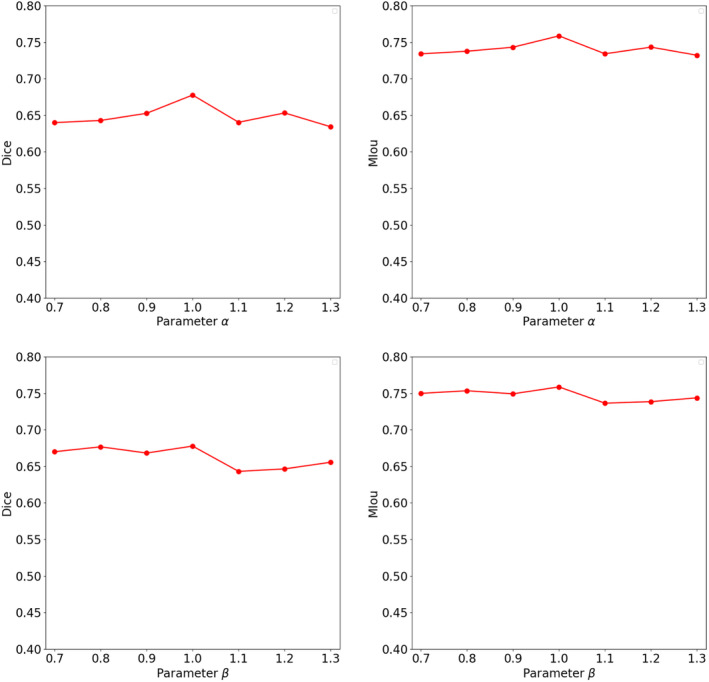
Sensitivity analysis for hyperparameters, *α* and *β*.

Continuing with our comparison, we evaluated our proposed method against existing techniques for generalizing NPC segmentation. Table 1 presents a comparison of our method's performance in terms of Dice and MIoU with existing works. Our method evidently outperforms the baseline in handling the generalization of NPC segmentation. To make the algorithm comparisons more intuitive, Figure 3 shows the relationship between test dice and the number of epochs for the different algorithms. To visually demonstrate the effectiveness of different algorithms, we illustrated in Figure 4 the outcomes of employing various segmentation methods on different cases and modalities. The significant performance superiority of MF‐Net clearly demonstrates its effectiveness for generalizing NPC MRIs segmentation to unseen domains. To assess the influence of the hypermeters α and β on our MF‐Net model. We adjust its value in {0.7, 0.8, 0.9, 1.0, 1.1, 1.2, 1.3} while fixing the other hyperparameter to the value used in the original MF‐Net model. Figure 5 illustrates that the performance of our proposed model remains stable across a range of values for these trade‐off hypermeters, indicating a low sensitivity to these hypermeters.

## 4 DISCUSSION

In clinical practice, the identification of NPC boundaries relies primarily on the experienced oncologists, particularly in delineating the region of interest of tumour. Due to the limitations of MR imaging, such as high variability, low contrast and discontinuous edges in soft tissue appearance, recognizing tumour boundaries faces a challenge. With the advancement of machine learning, many researchers have begun exploring automatic segmentation of tumour regions using machine learning methods, such as U‐Net^25^ and modified 3D,^26^ V‐Net,^27^ CA‐Net^28^ and MMFNet^29^ models and showed an excellent performance in medical image segmentation. In the segmentation of NPC based on MRI images, deep 3D CNN model, SSL‐based deep learning model and MMFNet were successively proposed and achieved a superior accuracy.^30^, ^31^ A large‐scale and multi‐centre study from Luo et al. proposed an augmentation‐invariant strategy to delineate the GTV for NPC MRI images and effectively reduced the performance gap between internal and external testing data.^32^

In most clinical cases, the task of collecting multi‐centre data is difficult due to the privacy principle. Therefore, data augmentation in a single domain is commonly used as a feasible approach to address domain generalization issues. Mixup^22^ enhances new samples by interpolating images and labels to combine two samples. Instead of using the entire object regions, CutMix^33^ cuts and pastes patches from one image onto the other image, along with the ground truth labels being mixed proportionally to the area of patches. To improve the generalization of ultrasound image segmentation networks, Bi et al.^23^ designed a segmentation network based on mutual information, distinguishing image anatomical and domain features and employing a cross‐reconstruction method to train the network. Zhou et al.^24^ designed a dual normalization model to simulate appearance variations that may occur in unknown target domains. It first uses nonlinear transformations to enhance the original images into source‐similar and source‐dissimilar images and then trains the model based on the dual normalization technique.

The few methods that have addressed domain generalization in the field of medical image segmentation are specific to certain diseases. Facing the task of MRI segmentation in NPC, these methods may suffer from the degradation of performance. Therefore, we first evaluated the performance of existing domain generalization methods on NPC MRI images and then designed more optimized algorithms specifically tailored to the characteristics of NPC images.

In our study, the proposed NPC segmentation network uses triple modalities as input. With the advantage of the complementarity of triple modalities, the delineation of the GTV of NPC presents the more precise tumour boundary compared to the single modality. In this study, we first used MRIs collected from NPC patients from two hospitals to evaluate the current state‐of‐the‐art domain generalization methods in the invisible domain generalization of NPC image segmentation. In response to the problem of low efficiency in current methods, MF‐Net is proposed to solve the problem of generalization in the field of NPC MRI imaging tumour segmentation. Specifically, in the frequency domain, low‐level features of different signals are mixed, and Fourier inverse transform is performed to the spatial domain. Then, model training is conducted based on meta‐learning and compared with existing state‐of‐art methods, proving that our method can better describe the nasopharyngeal tumour body area. From the data in Table 1, it can be seen that our approach's efficacy elevates the Dice coefficients of the three modalities by 3.8%, 8.7% and 3.5% correspondingly. Moreover, the Mean Intersection over Union (MIoU) values witness an escalation of 0.5%, 1.1% and 0.9%, respectively. These outcomes incontrovertibly substantiate the potency of our method in augmenting the proficiency of image segmentation algorithms.

The clinical advantages of modern radiation therapy techniques are closely related to contouring accuracy, dose consistency and plan delivery accuracy. Currently, these processes of segmentation of NPC performed by oncologists are extremely time‐consuming and empirical. Suboptimal tumour coverage and low‐quality radiotherapy plans are major factors contributing to disease recurrence and low survival rates. In this context, our designed method not only improves the effectiveness of tumour contour recognition but also reduces the time required for contouring compared to manual delineation by oncologists.

Upon analysing the data presented in Table 1, it becomes apparent that there are variations in the recognition outcomes for different signals. This disparity can be ascribed to the inherent advantage of the CE‐T1WI sequence in discerning neighbouring boundaries, encompassing microvasculature, thereby manifesting exceptional segmentation efficacy. When delineating the tumour region, clinicians tend to incorporate the contouring on the T1WI sequence to a certain extent, using the CE‐T1WI as a reference framework. Consequently, their data annotations amalgamate the distinctive features of both CE‐T1WI and T1WI images. Notably, our MF‐Net algorithm has demonstrated a conspicuous aptitude for identifying anatomical structures of T1WI. Conversely, the T2WI sequence exhibits the most lacklustre performance in lesion recognition, plausibly as a result of the signal distortions stemming from oedematous interference.

In summary, we first propose an effective domain generalization method, MF‐Net, using the imaging characteristics of MRI to perform amplitude mixing on MRI images in the frequency domain to evaluate its performance in MRI segmentation. MF‐Net can increase the diversity of samples without affecting the external appearance of tumours. Additionally, we combine this approach with meta‐learning of mixed features to enhance the generalization of the model. Compared with the existing state‐of‐the‐art works on addressing domain generalization, the MF‐Net demonstrates exceptional performance.

## AUTHOR CONTRIBUTIONS

**Yin Li:** Conceptualization (lead); data curation (equal); investigation (equal); project administration (equal); resources (lead); writing – original draft (equal); writing – review and editing (equal). **Qi Chen:** Data curation (equal); formal analysis (equal); funding acquisition (equal); supervision (equal); visualization (equal). **Hao Li:** Data curation (equal); formal analysis (equal); investigation (equal); software (equal); validation (equal). **Song Wang:** Data curation (equal); formal analysis (equal); software (equal); validation (equal); visualization (equal); writing – original draft (equal). **Nutan Chen:** Conceptualization (equal); data curation (equal); investigation (equal); methodology (equal); supervision (equal); validation (equal); writing – original draft (equal). **Ting Han:** Data curation (equal); formal analysis (equal); software (equal); validation (equal); visualization (equal); writing – original draft (equal). **Kai Wang:** Formal analysis (equal); software (equal); writing – original draft (equal). **Qingqing Yu:** Data curation (equal); investigation (equal); resources (equal). **Zhantao Cao:** Conceptualization (equal); formal analysis (equal); investigation (equal); methodology (equal); writing – review and editing (equal). **Jun Tang:** Conceptualization (equal); funding acquisition (equal); writing – review and editing (equal).

## FUNDING INFORMATION

This present study is supported by the Foshan Science and Technology Innovation Project (2220001003814) and the Second Affiliated Hospital of Anhui Medical University Clinical Research Development Program (2021LCYB10).

## CONFLICT OF INTEREST STATEMENT

The authors declare that they have no competing interests.

## CONSENT TO PARTICIPATE

Written informed consent was obtained from the individual(s) to publish any potentially identifiable images or data in this article.

## Data Availability

The datasets used and/or analysed during the current study are available from the corresponding author upon reasonable request.
